# Genome-wide identification, characterization, and expression patterns of the BZR transcription factor family in sugar beet (*Beta vulgaris* L.)

**DOI:** 10.1186/s12870-019-1783-1

**Published:** 2019-05-09

**Authors:** Wei Wang, Ya-Qing Sun, Guo-Long Li, Shao-Ying Zhang

**Affiliations:** Sugar Beet Physiological Research Institute, Inner Mongolia Agricultural University, Hohhot, China

**Keywords:** BZR transcription factor, Expression pattern, Genome-wide analysis, Sugar beet, Taproot development

## Abstract

**Background:**

BRASSINAZOLE-RESISTANT (BZR) family genes encode plant-specific transcription factors (TFs) that participate in brassinosteroid signal transduction. BZR TFs have vital roles in plant growth, including cell elongation. However, little is known about *BZR* genes in sugar beet (*Beta vulgaris* L.).

**Results:**

Therefore, we performed a genome-wide investigation of *BvBZR* genes in sugar beet. Through an analysis of the BES1_N conserved domain, six *BvBZR* gene family members were identified in the sugar beet genome, which clustered into three subgroups according to a phylogenetic analysis. Each clade was well defined by the conserved motifs, implying that close genetic relationships could be identified among the members of each subfamily. According to chromosomal distribution mapping, 2, 1, 1, 1, and 1 genes were located on chromosomes 1, 4, 5, 6, and 8, respectively. The *cis*-acting elements related to taproot growth were randomly distributed in the promoter sequences of the *BvBZR* genes. Tissue-specific expression analyses indicated that all *BvBZR* genes were expressed in all three major tissue types (roots, stems, and leaves), with significantly higher expression in leaves. Subcellular localization analysis revealed that Bv1_fxre and Bv6_nyuw are localized in the nuclei, consistent with the prediction of Wolf PSORT.

**Conclusion:**

These findings offer a basis to predict the functions of *BZR* genes in sugar beet, and lay a foundation for further research of the biological functions of *BZR* genes in sugar beet.

**Electronic supplementary material:**

The online version of this article (10.1186/s12870-019-1783-1) contains supplementary material, which is available to authorized users.

## Background

Brassinosteroids (BRs) are plant-specific hormones that participate in a wide range of developmental processes during the plant lifecycle [1]. With recent technological developments in biochemistry and molecular biology, great progress has been made in the study of BR signal transduction using different types of mutants [2–4]. Unlike animal steroid hormones, which directly target nuclear receptors, BRs bind to BRASSINOSTEROID-INSENSITIVE 1 (BRI1) [5], a membrane-localized receptor, and then target BRASSINAZOLE-RESISTANT (BZR) transcription factors (TFs) [6], which in turn regulate BR-responsive genes [7–9].

BZR TFs are key elements of BR signal transduction. In *Arabidopsis thaliana*, the *BZR* gene family includes BRASSINAZOLE-RESISTANT 1 (*BZR1*), BRI-EMS-SUPPRESSOR1 (*BZR2/BES1*), and *BZR1/2* Homologs 1–4 (*BEH1*–*BEH4*), which show high sequence identity with *BZR1/2* [10, 11]. *BZR1* and *BZR2/BES1* have roles as transcriptional repressors and activators, respectively [10, 12–14]. For instance, *BZR1* binds to the promoters of *CPD* and *DWF4* in vivo by identifying the sequence CGTG(T/C) G, ultimately suppressing transcription [12, 15, 16]. By contrast, *BES1* binds to E box (CANNTG) sequences in the promoters of BR-induced genes by recognizing a basic helix-loop-helix protein, BIM1 [10]. *BZR1* and *BZR2* exhibit protein sequence similarity of up to 88% [10, 17]. Chromatin immunosuppression quantitative PCR experiments have indicated that both *BZR1* and *BZR2/BES1* bind to the BR-repressed gene *DWF4* and BR-induced gene *SAUR-AC1* [18]. Moreover, *BZR2/BES1* binds to 18 of the 19 BZR1 binding sites. Studies indicate that BZR TFs may be involved in plant growth and development via the regulation of other TFs [11, 19]. Therefore, identifying new *BZR* genes from various plant species represents a reliable approach to obtain new insight into the *BZR* gene family.

Increasing crop yield and improving crop quality are two main goals in agricultural production. Among critically important plant steroid hormones, BRs are involved in a wide range of cellular responses, including cell elongation, tolerance to environmental stresses, and resistance to pathogens, through which they can also increase yields [20]. In addition, useful agricultural applications of BRs have been identified, including improving the yield and stress resistance of several major crops. For example, during the rapid leaf or root growth period, as well as the sugar storage period, BRs can increase the SPAD-based chlorophyll content of sugar beet (*Beta vulgaris* L.) and improve the net photosynthetic rate and stomatal conductance of leaves, ultimately improving production.

Beetroot is a crucial organ in sugar beet, a sugar-yielding crop that accounts for 30% of the global sucrose output [21]. Although numerous TFs have been studied in this plant, there is little research on the BZR family in sugar beet, in particular in relation to developmental functions.

Therefore, we performed a comparative genomic analysis of *BvBZR* genes to analyze this gene family in sugar beet comprehensively. First, *BvBZR* genes were identified according to published transcriptome analyses. A phylogenetic analysis and conserved domain sequence search were used to cluster the family into three groups. Next, *BvBZR* expression patterns in three major tissue types (roots, stems, and leaves) and in response to phytohormones were analyzed during the growth period using quantitative real-time reverse transcription (qRT)-PCR. In addition, we investigated the effects of *BvBZR* genes on beetroot development. These findings provide a basis for future research on the structure and functions of *BvBZR* genes, as well as identifying and characterizing *BZR* genes in other species. In addition, this study offers a theoretical basis for further work on the molecular mechanisms of beetroot growth.

## Results

### Identification of *BvBZR* genes

The hidden Markov model profile of the BES1_N domain was used to identify *BZR* genes in the sugar beet genome, from which six *BvBZR* genes were identified. Subsequently, the protein amino acid sequences of the respective genes were subjected to SMART analysis, and were consistent with previous findings. Because there are no reports of any *BZR* gene family members in sugar beet, *BvBZR* genes were provisionally named according to the sugar beet database as follows: *Bv5_cuzi*, *Bv_epwr*, *Bv1_fxre*, *Bv6_nyuw*, *Bv1_qnjn*, and *Bv_yfzt*.

### Conserved motifs and phylogenetic analysis of the BvBZR family

**Fig. 1:**
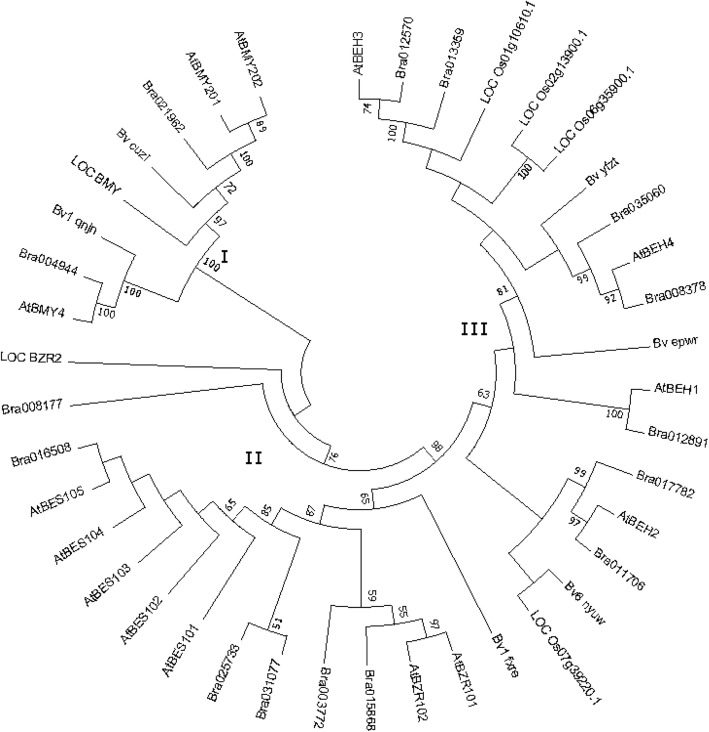
Phylogenetic analysis and the grouping of BZR family

To clarify the evolutionary relationship of the BZR family, we constructed phylogenetic trees based on amino acid sequences from 41 BZR family members from sugar beet, *A. thaliana*, rice, and Chinese cabbage (Fig. 1). BZR TFs were clustered into three groups containing 8, 15, and 18 members, respectively. Each BvBZR subunit was clustered with their possible homologs in *A. thaliana*. For instance, clade II contained AtBZR1 and AtBZR2/BES1, as well as *Bv1_fxre*, indicating that it had the highest homology with AtBZR1/2. Meanwhile, Bv6_nyuw had the highest homology with AtBEH2. Regardless of these associations, the functions of AtBEH4 and AtBEH2 have not been determined [10].

**Fig. 2:**
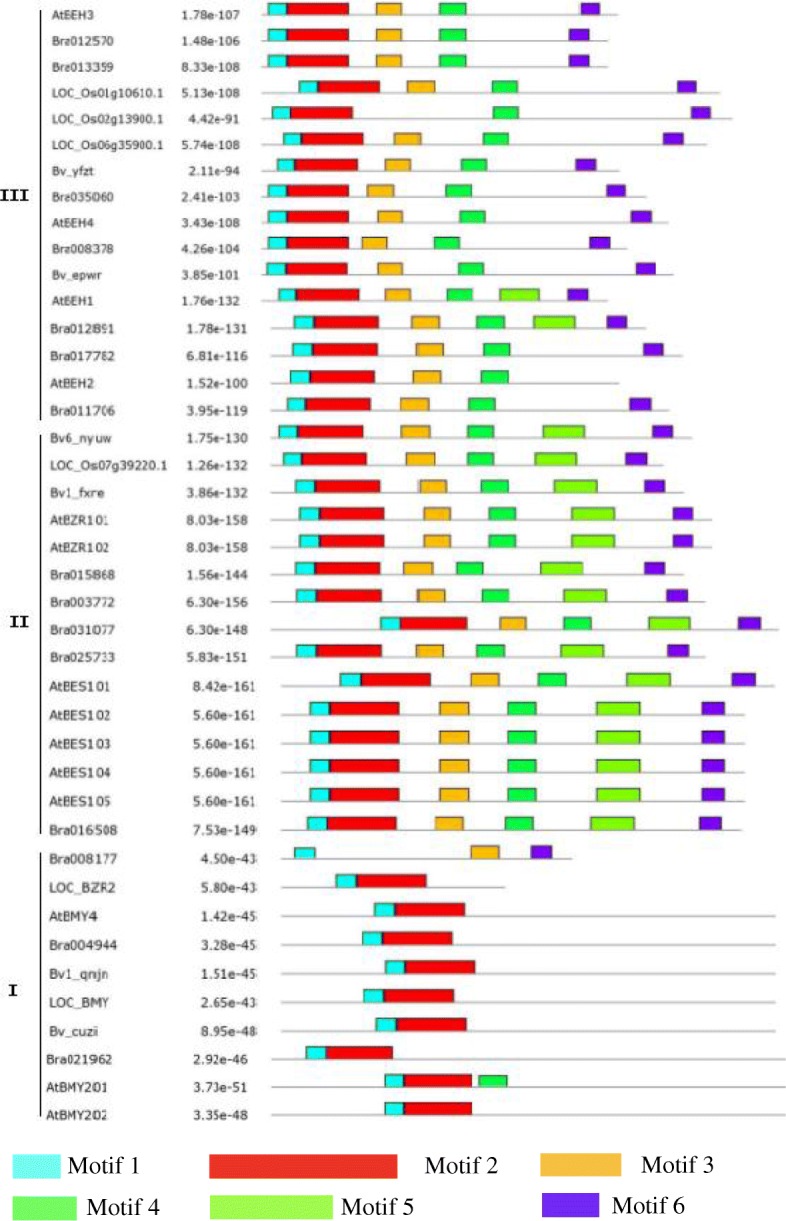
The distribution of conserved motifs for BZR proteins

To understand the diversity of BZR protein structures, MEME analysis was performed to predict the conserved motifs in the BZR family in sugar beet, *A. thaliana*, rice, and Chinese cabbage, which identified six conserved motifs. The BZR members were clustered into three subunits (clades I, II, and III) (Additional file 1; Fig. 2). Each clade shared similar motifs: clade III contained the conserved motifs 1, 2, 3, 4, and 6; clade II contained motifs 1, 2, 3, 4, 5, and 6; and clade I contained motifs 1 and 2. Despite overlaps, each clade had specific motifs; for example, only clade II contained motif 5, whereas motifs 3, 4, and 6 were found in all clades except clade I. Although *Bv_epwr*, Bv6_nyuw, and Bv1_fxre were distributed into different subunits, the same motifs were identified in these proteins, suggesting that they have similar functions. A similar association was observed for Bv1_qnjn and Bv5_cuzi.

### Chromosomal distribution and gene structures of *BvBZR* genes

**Fig. 3:**
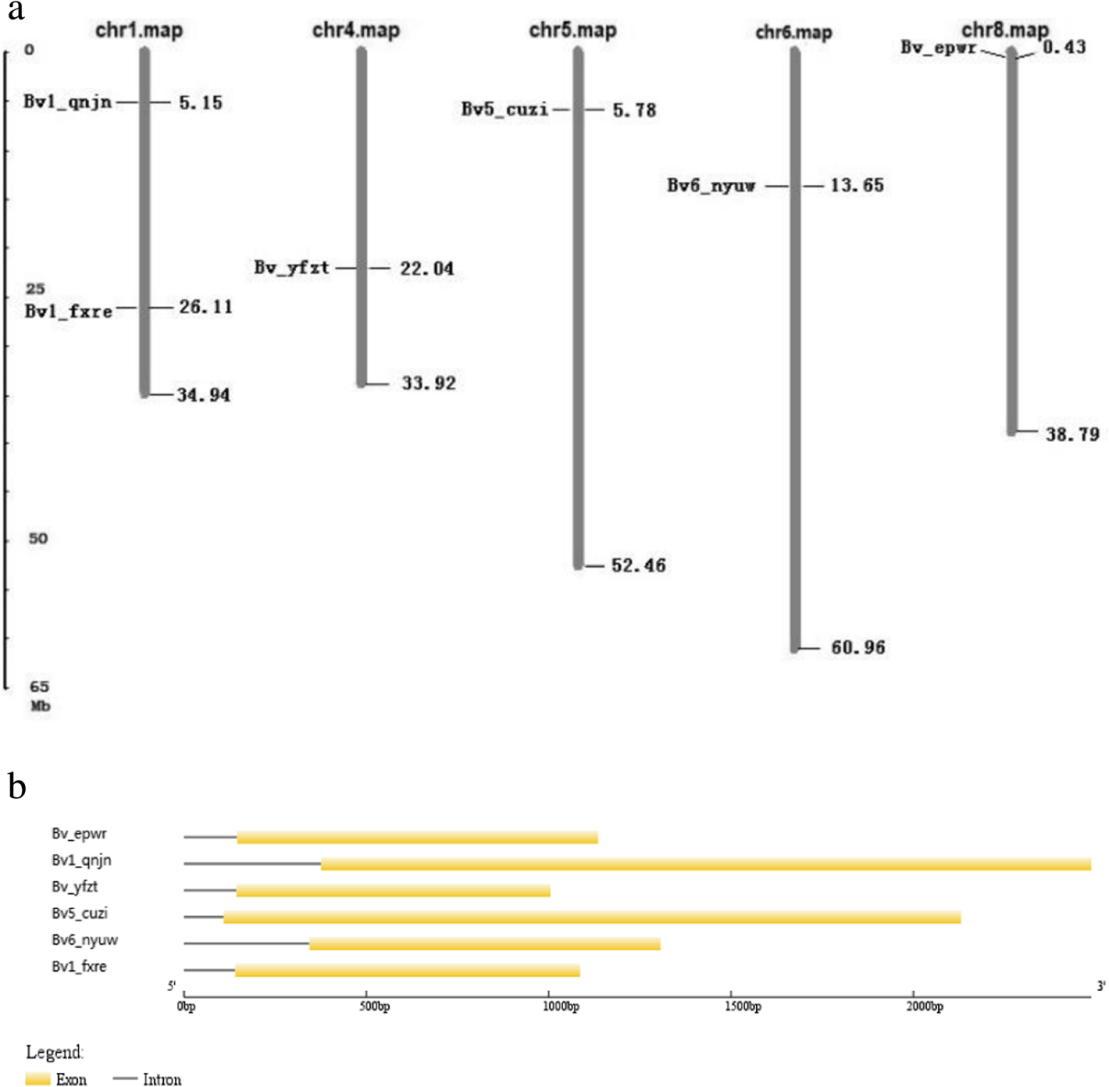
Chromosomal distribution and gene structures of *BvBZR* genes. **a** Distribution of the *BvBZR* genes on *Beta vulgaris* chromosomes. **b** Exon-intron structures analyses of *BvBZR* genes

The six identified *BvBZR* genes were mapped onto the five sugar beet chromosomes (Fig. 3a). Two *BvBZR* genes (*Bv1_qnjn* and *Bv1_fxre*) were located on chromosome 1, and the other four were equally distributed on chromosomes 4, 5, 6, and 8. An analysis of the *BvBZR* gene structures revealed that all members contained only one intron (Fig. 3b).

### Analysis of *cis*-acting elements in *BvBZR* gene promotors

**Fig. 4:**
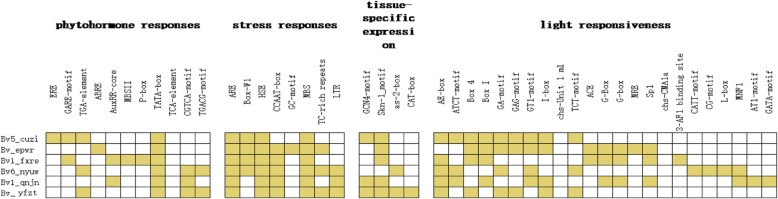
Analysis of *cis*-acting elements in *BvBZR* gene promotors

To investigate the *cis*-acting elements in the promoter regions of *BvBZR* genes, approximately 1500 bp of sequence upstream of the translation initiation site were analyzed using PlantCARE. In total, 63 *cis*-acting elements were found, which were related to phytohormone, stress, and light responses, as well as tissue-specific expression in the promoters of different *BvBZR* genes (Fig. 4). All *BvBZR* upstream sequences contained a putative TATA box. Light-responsive elements had the largest number of *cis*-acting elements, followed by stress-response elements, and finally phytohormone-responsive and tissue-specific-expression elements, for which several genes had only one element. Different genes included not only differing numbers of phytohormone-responsive elements, including *Bv1_fxre* (four elements) versus *Bv1_qnjn* (two elements), but also elements that responded to different types of phytohormones. For example, *Bv1_fxre* contained auxin- and gibberellin-responsive elements, however, *Bv_epwr* contained only an abscisic acid-responsive element. These findings suggest that different *BvBZR* genes have different regulatory effects during taproot growth by responding to different phytohormone signals.

In a further analysis of the types of phytohormone-responsive elements found in *BvBZR* gene promoters, four types of elements were found: gibberellin, abscisic acid, auxin, and methyl jasmonate. Gibberellin- and auxin-responsive elements were found to promote growth, while the others facilitated maturation. Therefore, *Bv1_fxre*, which contained a gibberellin-responsive element, was more likely to promote sugar beet growth, whereas *Bv_epwr*, which contained only an abscisic acid-responsive element, likely promoted maturation.

### *BvBZR* gene expression patterns during vegetative growth

**Fig. 5:**
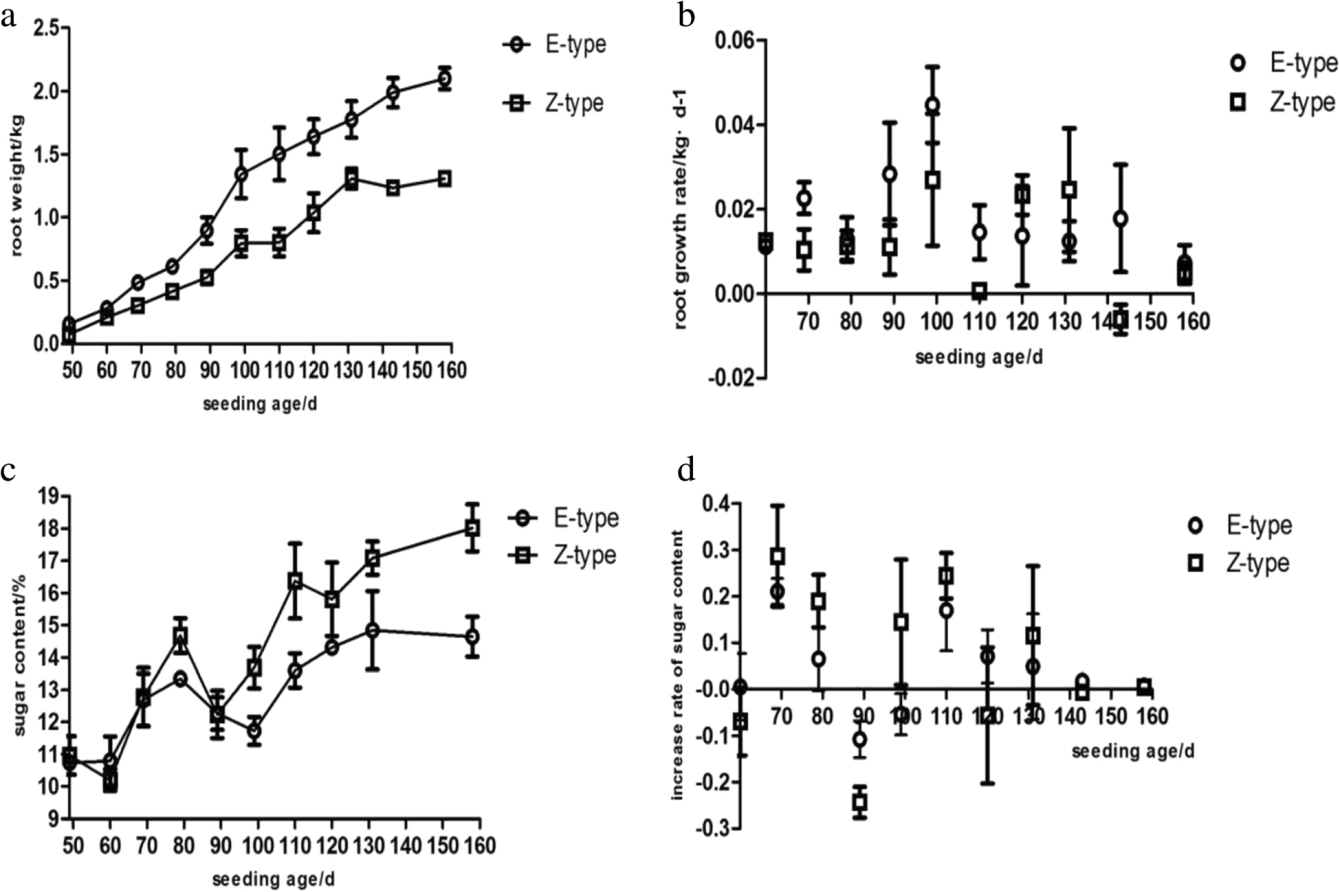
Taproot growth rhythm of sugar beet. The E-type cultivar SD13829 and the Z-type cultivar BS02 were grown in field. **a** The growth curve of taproot. **b** The growth rate of taproot. **c** The sugar content curve of taproot. **d** The increase rate of sugar content of taproot

Figure 5 illustrates the growth characteristics of beetroot. During the whole vegetative period, the root weight of the Ertrag-type (E-type) cultivar was heavier than that of the Zucker-type (Z-type) cultivar. The root growth rate reached a maximum in both cultivars in seedlings aged 99 days. Over the course of the vegetative period, Z-type had a greater sugar content than E-type, although the sugar accumulation rate reached a maximum in both cultivars in seedlings aged 69 days.

**Fig. 6:**
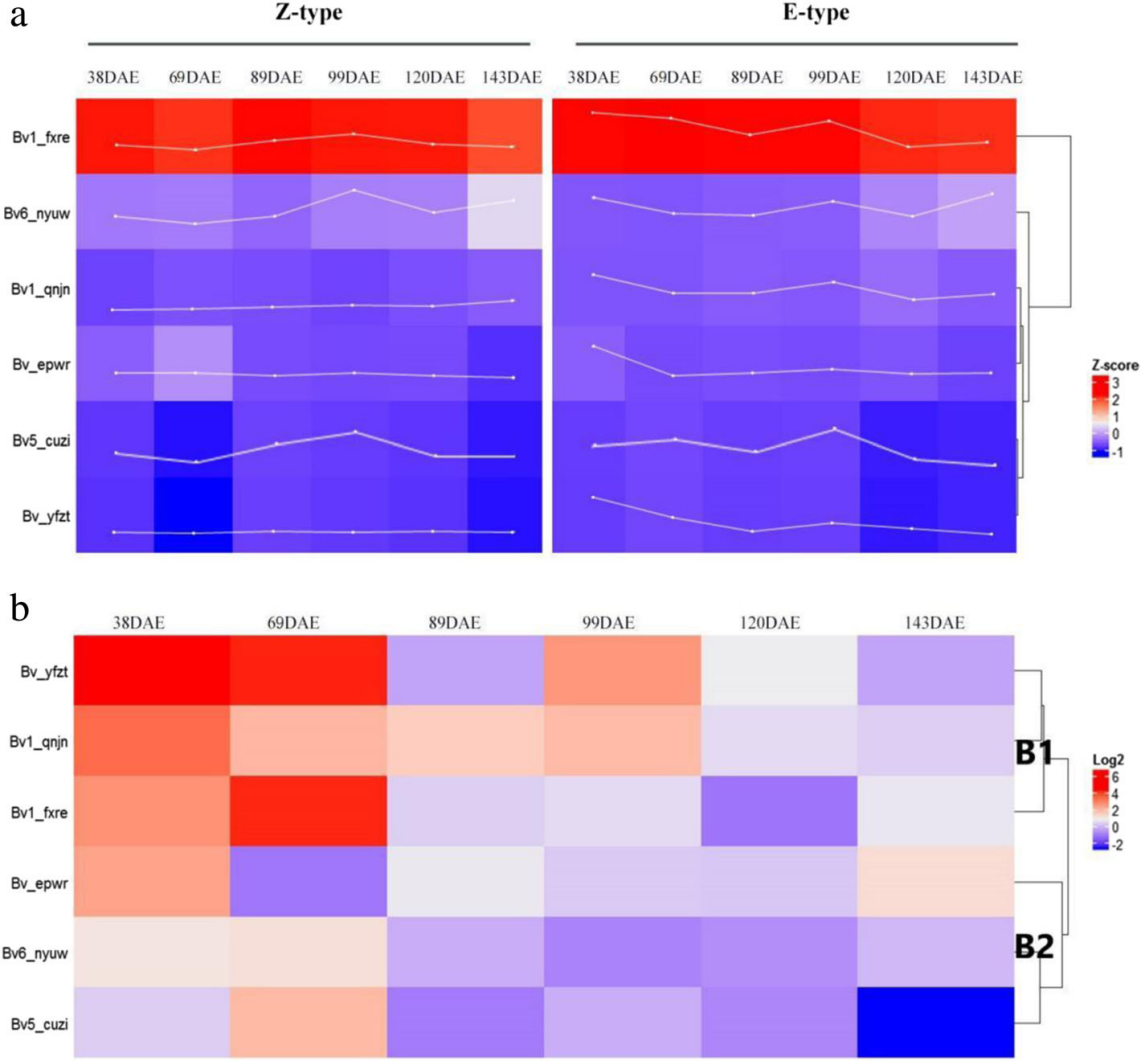
The expression patterns of *BvBZR* genes. **a** The clustering anslysis for expression pattern of *BvBZR* family gene in taproot development. The expression level of *BvBZR* genes in the taproot of E-type cultivar SD13829 and Z-type cultivar BS02 at 38, 69, 89, 99, 120 and 143 DAE. **b** The different expression of *BvBZR* genes in the taproot of E-type cultivar SD13829 compared to that in the taproot of Z-type cultivar BS02 at 38, 69, 89, 99, 120 and 143 DAE

Gene expression patterns are often correlated with gene function. Therefore, a heatmap was created according to the four periods related to the growth characteristics of sugar beet (Additional files 2 and 3; Fig. 6). The expression levels of all *BvBZR* genes were up-regulated in both cultivars in seedlings aged 99 days, when the root growth rate reached the maximum in both cultivars. In particular, *Bv5_cuzi* expression peaked on day 99. However, except for *Bv1_qnjn*, the other five *BvBZR* genes exhibited decreased expression levels in Z-type seedlings aged 69 days. Moreover, *Bv1_fxre* expression was significantly higher than all other *BvBZR* genes during the whole period (Fig. 6a). To gain greater insight into their potential functions, a correlation analysis of *BvBZR* gene expression with beetroot growth rate and sugar accumulation rate was performed. Positive correlations were observed between *Bv5_cuzi*, *Bv1_fxre*, and *Bv_yfzt* with root growth (Table 1). However, the other genes exhibited negative correlations with root growth, especially *Bv1_qnjn*, which exhibited a significant negative correlation. Interestingly, *Bv1_qnjn* was positively correlated with sugar accumulation rate, whereas the other genes, particularly *Bv5_cuzi*, showed negative correlations.

**Table 1:** Correlation analysis of *BvBZR* gene expression with beetroot growth rate and sugar accumulation rate

	*Bv5_cuzi*	*Bv_epwr*	*Bv1_fxre*	*Bv6_nyuw*	*Bv1_qnjn*	*Bv_yfzt*
Root growth rate	0.485	−0.685	0.826	−0.425	−0.999*	0.451
Increased rate of sugar accumulation	−0.998*	−0.883	−0.641	−0.721	0.968	−0.658

* indicate a significant difference at *P* < 0.05

To investigate the variations in *BvBZR* genes between the two investigated cultivars, a cluster analysis was conducted, which divided the *BvBZR* genes into two groups (Fig. 6b). In seedlings aged 38 and 69 days, the expression levels of B1 subgroup genes were higher in E-type than in Z-type. At the peak root growth rate, in seedlings aged 99 days, the two cultivars exhibited significant differences in gene expression, and B2 subgroup gene expression was lower in E-type than in Z-type. In seedlings aged 120 days, when the root growth rate was low, E-type plants exhibited lower expression of all *BvBZR* genes than Z-type, except for *Bv_yfzt*, which showed no differences between the two cultivars.

### *BvBZR* gene expression patterns in sugar beet tissues

**Fig. 7:**
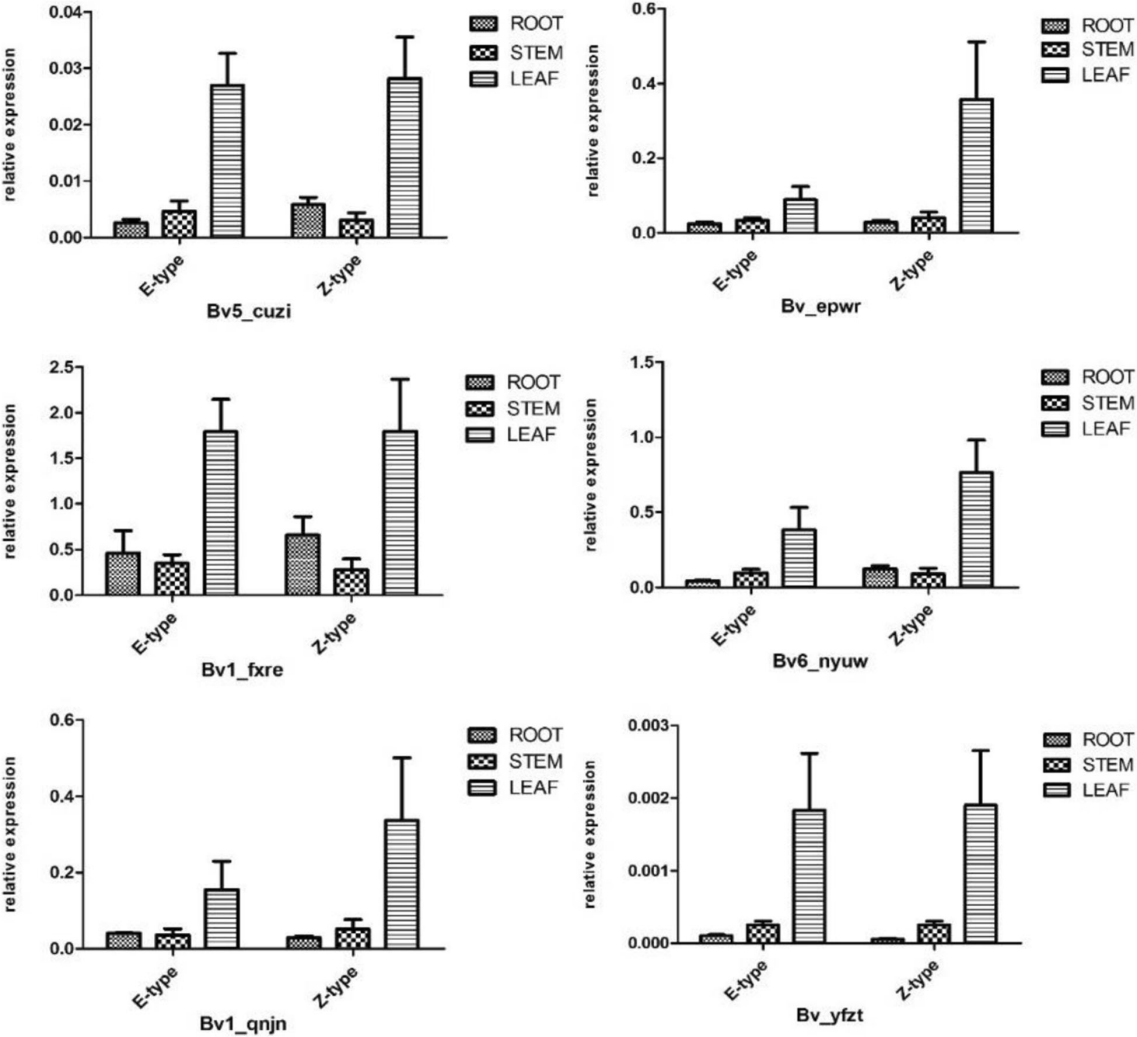
The analysis of organ specific expression for *BvBZR* genes

To obtain greater insight into the potential functions of *BvBZR* genes, we analyzed the expression patterns of *BvBZR* genes during the fastest root growth rate period (seedling age 99 days) in root, stem, and leaf tissues in both E-type and Z-type cultivars (Additional file 4). All six genes were expressed in all three tissue types, albeit with varying expression patterns, although the transcript abundances of certain genes in a few tissues were very low (Fig. 7). Almost all *BvBZR* genes were detected with the highest expression in leaves, compared to other tissues. Moreover, *Bv1_fxre* expression was higher in root than in stem, suggesting that it may have an important role in root growth in sugar beet.

### *BvBZR* gene expression patterns in response to phytohormones

**Fig. 8:**
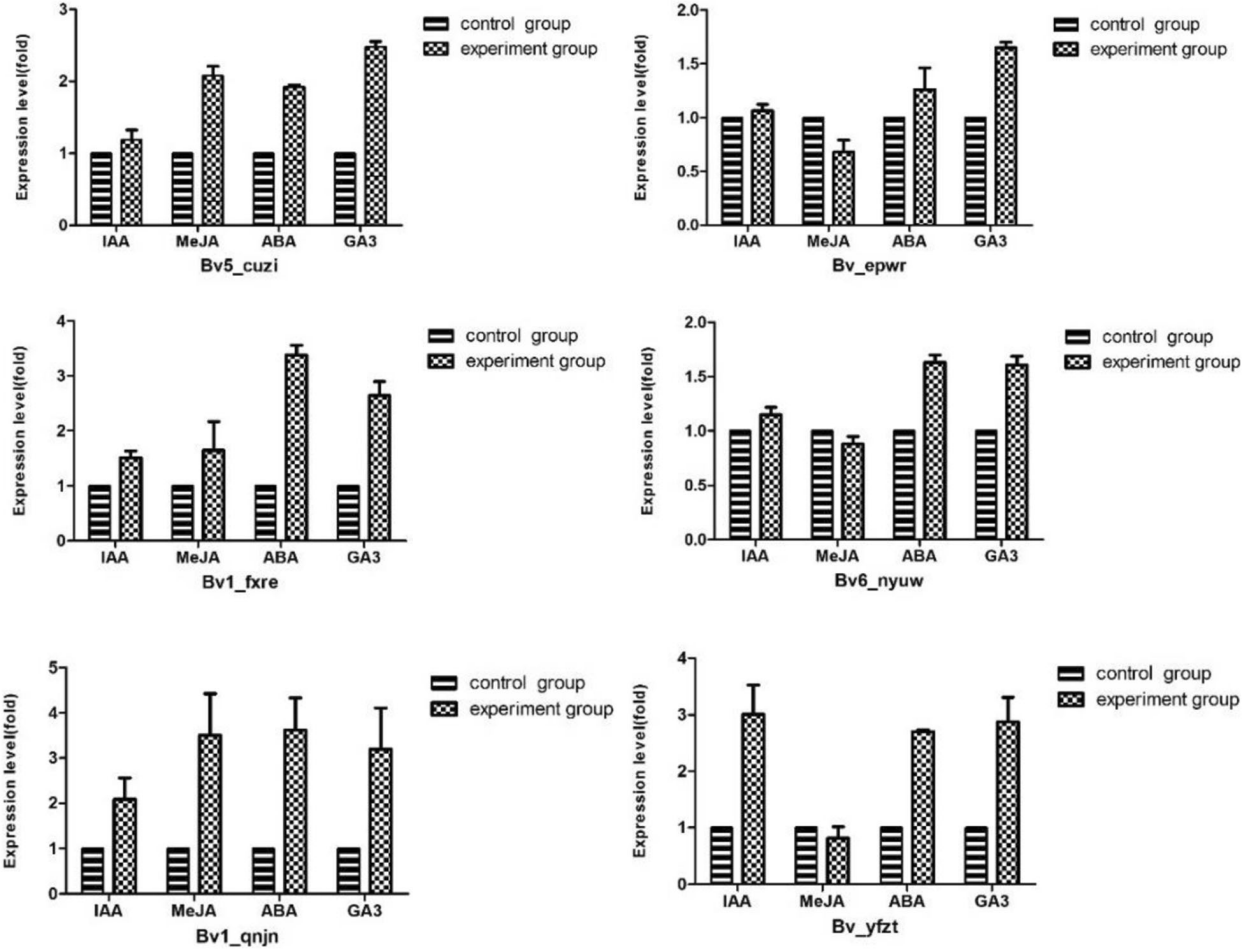
Response of *BvBZR* genes to several phytohormones. The seedling age was 69 days. The exogenous GA_3_ (80 mg/L), ABA (10 mg/L), IAA (0.5 mg/L) and MeJA (200 μmol/L) were sprayed to the beetroot, the control group was sprayed with water

To investigate whether the expression levels of *BvBZR* genes are regulated by exogenous phytohormones in sugar beet, the beetroots were sprayed with indoleacetic acid (IAA), abscisic acid (ABA), Methyl jasmonate (MeJA), and gibberellin (GA_3_). As shown in Fig. 8, the expression level of *Bv_yfzt* increased three-fold after spraying with IAA, whereas *Bv_epwr* showed higher expression in response to ABA. Moreover, *Bv6_nyuw* was upregulated in response to GA_3_, which is consistent with the analysis of *cis*-acting elements in *BvBZR* gene promotors. The expression level of *Bv_epwr* was suppressed after spraying with MeJA, whereas the expression level of *Bv1_qnjn* increased, suggesting that *Bv_epwr* and *Bv1_qnjn* play distinct roles in response to MeJA.

### Subcellular localization of the BvBZR::sGFP fusion protein

**Fig. 9:**
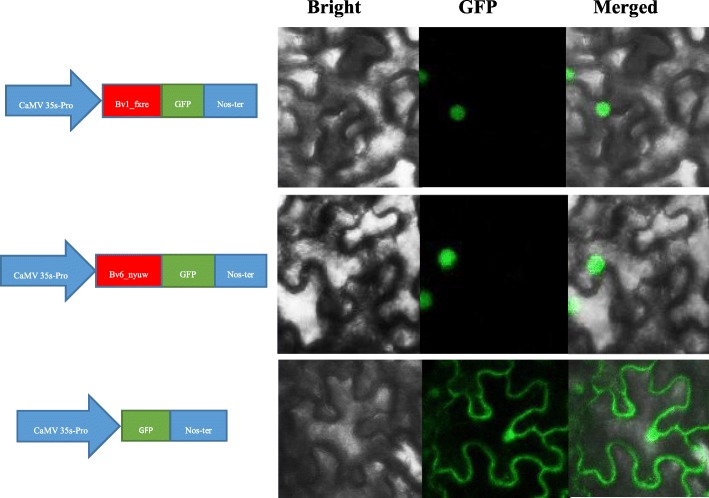
Subcellular localization of *Bv1_fxre* and *Bv6_nyuw*

To clarify the subcellular localization of *BvBZR* genes, the Wolf PSORT software was used, which predicted that the majority of *BvBZR* genes are localized in the nucleus (Additional file 5). To verify this result, the coding regions of *Bv1_fxre* and *Bv6_nyuw* were fused into the GFP binary vector, and GFP fluorescence was then expressed in *Nicotiana benthamiana* leaves. A strong fluorescent signal was observed in the nuclei (Fig. 9). However, the GFP signal was distributed throughout the cell with the 35S::GFP proteins. The results were consistent with the prediction made by Wolf PSORT.

## Discussion

The BZR family is an important TF family in plants, with a variety of roles in plant growth. Based on the completed whole-genome sequences, 14, 15, and 52 *BZR* genes have been identified in *A. thaliana* [12], Chinese cabbage [22], and legumes [23], respectively. In comparison, the sugar beet *BZR* gene family contains far fewer genes. To investigate the evolutionary relationship of BZR family members among species, we included BZR proteins from *A. thaliana*, rice, and Chinese cabbage in the conserved motif identification and phylogenetic analysis. The phylogenetic trees in the present study are similar to that in a study of Chinese cabbage [22]. The phylogenetic analysis divided *BvBZR* genes into three subgroups, where clade I contained *Bv5_cuzi* and *Bv1_qnjn*, clade II included *Bv1_fxre*, and clade II included all other genes. The conserved motif identification yielded the same results, except for *Bv6_nyuw*, which was clustered into clade II. The phylogenetic analysis implied that most *BZR* genes were derived from a common ancestor. In clade II, *Bv1_qnjn* was located in a separate subclade from *AtBZR1/2*, *LocBZR1/2*, and *BraBZR1/2*. In clade I, *Bv1_qnjn* was clustered into the same subclade as *Bra004944* and *AtBMY4*. These results suggest that *BZR* genes have a unique role in determining specific traits and functions in *A. thaliana*, rice, Chinese cabbage, and sugar beet.

To assess the potential functions of *BZR* genes in beetroot development, the functions of homologous genes in model plants were assessed. The phylogenetic tree constructed from 41 BZR proteins revealed that the protein Bv1_fxre was closely related to the *A. thaliana* homologs (Fig. 1). Moreover, an analysis of *cis*-acting elements in the promoters demonstrated that Bv1_fxre may have a role in promoting growth. Cell type-specific expression of a constitutively active form of BZR1 confirmed that high levels of BZR1 were required for normal cell behavior in the elongation zone [24]. Reductions in BES1 and BZR1 led to a semidwarf phenotype, and dominant mutations in BES1 and BZR1 resulted in distinct phenotypes in plants grown under light [10, 21, 25]. Finally, another study revealed that BZR1 synergized with gibberellin to regulate cell elongation [14, 26–28].

In seedlings aged 99 days, the Z-type and E-type cultivars exhibited significant differences in *Bv_yfzt* expression (Fig. 6b). Moreover, a positive correlation was observed between *Bv_yfzt* expression and root growth rate (Table 1), suggesting that Bv_yfzt has a role in beetroot growth. In seedlings aged 69 days, *Bv_epwr* was expressed more highly in the Z-type cultivar (Fig. 6b), consistent with the sugar accumulation rate (Fig. 5d), indicating that Bv_epwr may have a role in sugar accumulation within beetroot.

## Conclusion

In this study, we performed a genome-wide identification and characterization of the phylogenetic relationship, gene structure, chromosomal location, and promoter sequence analysis of *BZR* genes in sugar beet. Expression analyses revealed that all six *BvBZR* members were expressed more highly in leaves than in roots. Moreover, the findings suggest that *Bv1_fxre* and *Bv_yfzt* have roles in taproot development, whereas *Bv_epwr* has a role in sugar accumulation in the taproot. This is the first report to identify *BZR* genes in sugar beet, and provides a foundation to facilitate further research on the biological functions of the BZR family in sugar beet.

## Methods

### Plant materials and growth conditions

Two sugar beet cultivars were used in this study: E-type SD13829, which was provided by Strube (Söllingen, Germany), and Z-type BS02, which was bred in our laboratory. Seeds were cultivated at the Inner Mongolia Agricultural University farm (Hohhot, Inner Mongolia, China; 40°52′54″N, 111°43′53″E). Beginning on day 38 after emergence, the roots, stems, and leaves were collected simultaneously every 10 days. Three biological replicates were included for each cultivar during tissue collection. All samples were immediately frozen in liquid nitrogen and stored at − 80 °C until further analysis.

### Identification of *BvBZR* genes in the sugar beet genome

We used a hidden Markov model profile [29], using the BES1_N (PF05687) conserved domain sequence downloaded from the Pfam database (http://pfam.xfam.org) as the query, to identify all *BvBZR* sequences from the sugar beet databases RefBeet-1.1 and RefBeet-1.2 (http://bvseq.molgen.mpg.de/Genome/Download/index.shtml) using the BLASTp program. Furthermore, the 14 reported *A. thaliana* BZR protein sequences from the Arabidopsis Information Resource (https://www.arabidopsis.org/) also served as query sequences. The BLASTn program was applied using an E-value cutoff of 1.0 × 10^− 5^ to identify all *BvBZR* genes in the sugar beet genome. To ensure the validity of the identified *BvBZR* genes, the amino acid sequences of the respective proteins were checked for conserved domains using the Simple Modular Architecture Research Tool (SMART; http://smart.embl-heidelberg.de).

### Conserved motif identification and phylogenetic analysis of BZR proteins

Conserved motifs in the full-length amino acid sequences of BvBZR proteins were tested using MEME [2]; the number of motifs was set to six, and the default settings were applied to all other parameters.

The identified BvBZR domains from sugar beet, *A. thaliana*, rice, and Chinese cabbage were compared using Cluster-X 2.0. The phylogenetic tree was built using MEGA7.0. The neighbor-joining method was applied to construct different BZR TF trees using the pairwise deletion option. Bootstrapping was performed 1000 times to obtain support values for each branch.

### Chromosomal locations and gene structures of *BvBZR* genes

Information on the *BvBZR* genes, including chromosomal location and DNA and cDNA sequences, was obtained from the sugar beet database. MapInspect was used to map the distribution of the *BvBZR* genes. The exon/intron gene structures were constructed using the Gene Structure Display Server tool [15].

### Subcellular localization analysis

The *Bv1_fxre* and *Bv6_nyuw* open reading frames, excluding the termination codons, were amplified using specific primers. Amplified Bv1_fxre and Bv6_nyuw DNA were digested with *Bam*HI/*Sal*I and *Kpn*I/–*Xba*I restriction enzymes, respectively, and then inserted into the *Bam*HI/*Sal*I- and *Kpn*I/*Xba*I-digested pCAMBIA1300-35S-GFP vectors, respectively, to produce pCAMBIA1300-35S-FXRE::GFP and pCAMBIA1300-35S-NYUW::GFP. Both recombinant plasmids were transformed into *Agrobacterium tumefaciens* strain GV3101. Transient transformation of *N. benthamiana* epidermal cells with GV3101 carrying the fusion constructs was performed as described previously. GFP fluorescence was observed using an Airyscan confocal laser scanning microscope (ZEISS LSM 880, Carl Zeiss, Jena, Germany).

### Promoter sequence analysis of *BvBZR* genes

Approximately 1500 bp of sequence upstream from the start codon (ATG) in the *BvBZR* genes was determined as the regulatory promoter region from the sugar beet database. Subsequently, the promoter sequences were analyzed using the PlantCARE database (http://bioinformatics.psb.ugent.be/webtools/plantcare/html/).

### Measurement of the sugar beet response to exogenous phytohormones

BS02 plants were grown in soil for 76 days and then sprayed with GA_3_ (80 mg/L), ABA (10 mg/L), IAA (0.5 mg/L), MeJA (200 μmol/L), or water on the beetroot for 3 h. Roots were collected from three biological replicates. All samples were immediately frozen in liquid nitrogen and stored at − 80 °C until further analysis.

### qRT-PCR

Total RNA was extracted from each sample using TRIzol, under the guidance of Dr. Han Xiaomin. cDNA was diluted 16-fold before using as the template. qRT-PCR was performed using the CFX96 real-time PCR system (Bio-Rad, Hercules, CA, USA) in a 20-μL reaction containing 10 μL iTaq Universal SYBR Green Supermix (Bio-Rad), 0.5 μL each primer (10 μM), 2 μL cDNA template, and 7 μL double-distilled H_2_O. The PCR program was as follows: 95 °C for 2 min, followed by 40 cycles of 95 °C for 10 s, 55 °C for 10 s, and 72 °C for 30 s. After every reaction, a melting curve analysis was conducted to confirm that only one product was amplified and detected. The primer sequences are listed in Additional file 2: Table S1. The cycle threshold (C_t_) values were used to calculate fold-change differences in expression. Relative expression levels were determined using the 2^−△△Ct^ method [30]. All qRT-PCR experiments included three biological replicates, each with three technical replicates. The relative expression levels were then used to analyze *BvBZR* expression patterns during taproot growth and were mapped using Heatmap Illustrator (ver. 1.0.1; https://hemi.biocuckoo.org/). A correlation analysis of root, stem, and leaf gene expression levels with respect to beetroot growth rate and sugar accumulation was performed using SPSS (ver. 18.0).

## Additional files

Additional file 1: Distribution of conserved motifs in different protein families. Motif analysis was performed online by MEME; up to 6 motifs were permitted. (PDF 376 kb)

Additional file 2: **Table S1.** Primer sequences used for qRT-PCR. (XLSX 9 kb)

Additional file 3: **Table S2.** The expression level of *BvBZR* genes in two cultivars. (XLSX 10 kb)

Additional file 4: **Table S3.** The expression level of *BvBZR* genes in different tissues in two cultivars. (XLSX 8 kb)

Additional file 5: **Table S4.** Subcellular localization prediction programs for BvBZR. (XLSX 9 kb)

## Abbreviations

ABA: Abscisic acid
BRI1: BRASSINOSTEROID-INSENSITIVE 1
BRs: Brassinosteroids
BZR: BRASSINAZOLE-RESISTANT
*BZR2/BES1*: BRI-EMS-SUPPRESSOR1
DAE: Days after emergence
E-type: Ertrag-type
GA_3_: Gibberellin
IAA: Indoleacetic acid
MeJA: Methyl jasmonate
qRT-PCR: Quantiative real-time reverse transcription PCR
TFs: Transcription factors
Z-type: Zucker-type
