# Supplementary figures and images for: Genome-wide identification, characterization, and expression patterns of the BZR transcription factor family in sugar beet (Beta vulgaris L.)
Source: BMC Plant Biol. 2019 May 9;19:191. doi: 10.1186/s12870-019-1783-1 (PMC6506937; doi:10.1186/s12870-019-1783-1)

Motif 1

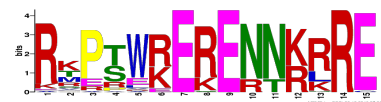

Motif 2

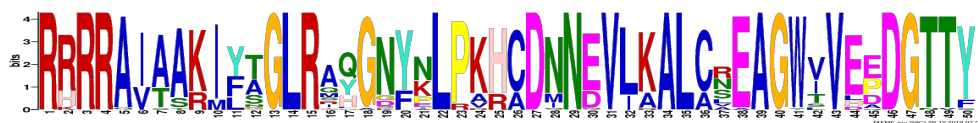

Motif 3

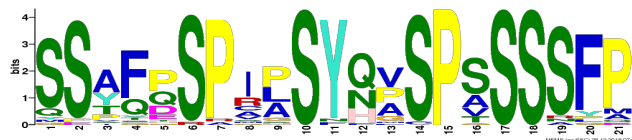

Motif 4

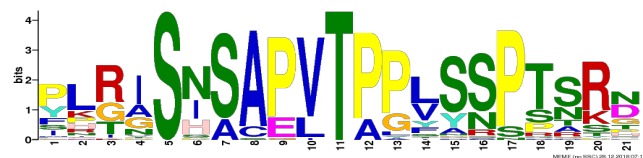

Motif 5

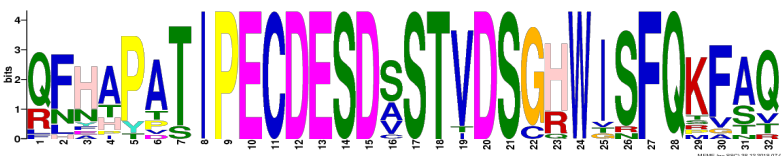

Motif 6

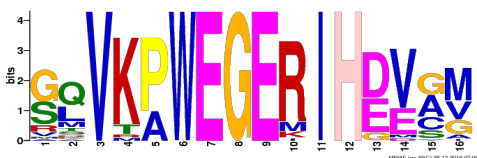

Supplement: Supplementary file 1 — Distribution of conserved motifs in different protein families. Motif analysis was performed online by MEME; up to 6 motifs were permitted. (PDF 376 kb) [file 12870_2019_1783_MOESM1_ESM.pdf]
